# Two successful pregnancies following fertility preservation in a patient with anaplastic astrocytoma: a case report

**DOI:** 10.1186/s12885-018-4472-9

**Published:** 2018-05-09

**Authors:** Alexandra Peyser, Sara L. Bristow, Avner Hershlag

**Affiliations:** 1 Department of Obstetrics and Gynecology, Northwell Health, Division of Reproductive Endocrinology, 300 Community Drive, Manhasset, NY 11030 USA; 2 Hofstra-Northwell School of Medicine, 500 Hofstra Blvd, Hempstead, NY 11549 USA

**Keywords:** Fertility preservation, Anaplastic astrocytoma, Glioma, Brain cancer

## Abstract

**Background:**

Astrocytomas are the most common malignant glial tumors. With improved prognosis, it is possible for patients to pursue pregnancy post-treatment. However, with potential gonadotoxicity of oncology treatments, fertility preservation prior to chemotherapy and/or radiation therapy should be considered. This requires close collaboration between the oncologist and reproductive endocrinologist. To our knowledge this is the first report of successful pregnancies following fertility preservation for AA.

**Case presentation:**

33-year-old nulligravid woman with newly diagnosed anaplastic astrocytoma (AA; WHO grade III, IDH1-negative) sought fertility preservation. Prior to chemotherapy and radiation for AA, the patient underwent in vitro fertilization (IVF) for fertility preservation, resulting in 8 vitrified embryos. Following chemo-radiation, the patient underwent two rounds of frozen embryo transfers (FET), each resulting in a successful singleton pregnancy.

**Conclusion:**

This case illustrates the realistic possibility, in carefully selected patients with brain tumors, of oocyte or embryo cryo-preservation prior to chemo-radiation and subsequent pregnancies.

## Background

Astrocytomas are the most common malignant glial tumors originating from small star- shaped glial cells (astrocytes) within the central nervous system. Anaplastic astrocytomas (AA) are defined as grade III glial tumors according to the WHO 2000 classification [1]. The incidence of AA is approximately 0.48 per 100,000 person/years. They occur more often in younger adults ages 30–50 and account for 17% of primary malignant brain tumors [2]. Prognosis in historical studies, which include both IDH (Isocitrate dehydrogenase)-mutant and IDH-wild type AAs, ranges from 3 to 5 five-year-survival. Prognosis is better for a genetically-defined subset of IDH-mutant tumors, with a median survival closer to 10 years [3]. The mainstay of therapy is surgery followed by radiotherapy. Multiple protocols, including various combinations of high dose radiotherapy, chemotherapy, alternative fraction regimens, heavy particle treatment, interstitial brachytherapy and radiosurgery have been proposed to extend survival [3].

Determining the safety of fertility preservation and subsequent pregnancy after treatment of gliomas is difficult due to the lack of data in the literature. Most studies have been done in patients where the glioma was diagnosed during pregnancy; in these cases there have been reports of changes in the growth of the tumors throughout the pregnancy [4–8]. Significantly, it seems that the same hormones and growth factors required for fetal development may also enhance tumor growth [9]. Currently, no guidelines exist for the medical management and treatment of gliomas diagnosed prior to or during pregnancy. Therefore, it is recommended that women with treated gliomas who want to pursue pregnancy should be followed by a high-risk obstetrician as well as a neuro-oncologist and monitored throughout pregnancy.

## Case presentation

A 33-year-old nulligravid woman with newly diagnosed AA (WHO grade III, IDH1 negative) presented to our office for fertility preservation. The patient had undergone a craniotomy with complete resection of her right parietal lobe tumor one month prior, and was scheduled to start chemotherapy and radiation in the next month. Her neuro-oncologist recommended that she undergo fertility preservation prior to chemo-radiation. The fertility preservation did not delay the anticipated start of her chemo-radiation treatment.

The patient had no significant medical or gynecological history. On physical exam, the patient was a healthy-appearing woman. She had left lower extremity weakness and instability. Transvaginal ultrasound demonstrated a normal-appearing uterus and ovaries bilaterally. A dominant follicle was noted on her right ovary; therefore, it was decided to administer HCG 10,000 IU at the time of her presentation to trigger ovulation, thus enabling the initiation of gonadotropins two weeks later. The patient had a high antral follicle count (6 on right, 7 on left).

The patient received low dose gonadotropins: 1 ampule of Human Menopausal Gonadotropin (Menopur®, Ferring Pharmaceuticals, Parsippany, NJ, USA), 75–187.5 IU of FSH (Gonal F®, EMD Serono, Rockland, MA, USA) for 10 days and cetrorelix acetate (Ganirelex®, GnRH antagonist, EMD Serono, Rockland, MA, USA) for the last 6 days. Final oocyte maturation was triggered with Lupron Luprolide Acetate (Lupron®, GnRH agonist, SANDOZ Pharmaceuticals, Princeton, NJ, USA) 40u. Twelve oocytes were retrieved transvaginally under ultrasound guidance. Eight embryos developed and were vitrified in liquid nitrogen (6 on day 3 and 2 on day 5 post-retrieval).

The patient returned to our Center one year later after she was cleared by her neuro-oncologist following the completion of chemotherapy and radiation. The patient had 6 weeks of radiation therapy with Temozolomide (Temodar®, Merck&Co, Inc., Whitehouse Station, NJ, USA) followed by 6 months of maintenance dose. Her last dose of chemotherapy was one month prior to returning to the office. The patient had maintained regular cycles post chemotherapy. The patient underwent a frozen-thaw natural cycle embryo transfer of a single day-3 embryo with vaginal progesterone (Crinone®, Actavis, Parsippany, NJ, USA) luteal phase support. The patient remained on Keppra® 500 TID (levetiracetam, UCB Pharmacueticals, Brussels, Belgium) and Lactulose throughout the pregnancy. A viable singleton pregnancy was seen on ultrasound 1 month later. The patient delivered a healthy female baby weighing 7lbs 5 oz. at term.

The patient returned two years later desirous of another pregnancy. Her neurological status had been stable, was tumor free and was cleared by her oncologist to conceive again. This time the patient was treated with Estrace® (estradiol, Warner Chilcott, Rockaway, NJ, USA) 6 mg a day and underwent a frozen-thaw cycle with a single day-5 blastocyst transferred. The patient conceived with a viable singleton pregnancy and delivered a healthy male at term weighing 6lbs.

Throughout the patient’s treatment regimen for fertility preservation and frozen embryo transfers, no adverse or unanticipated events were encountered.

## Discussion and conclusions

Women diagnosed with gliomas during child-bearing years may undergo fertility preservation prior to receiving chemotherapy and radiation to harvest oocytes and freeze them or freeze embryos if they have a partner, since their postoperative treatment, especially chemotherapy, is potentially gonadotoxic and may render them sterile. Studies have shown that the risk of ovarian failure as a result of chemotherapy varies based on both the drugs used as well as the patient’s age [10, 11]. Temozolomide (Temodar®, Merck&Co, Inc., Whitehouse Station, NJ, USA) is an alkylating agent, and while the effects of other alkylating agents used for chemotherapy on fertility have been studied, little is published about the gonadotoxicity of temozolomide in females. A handful of small studies have shown that fertility potential is affected in males [12, 13], with one case resulting in fathering a healthy child after treatment with temozolomide [13]. A study from France followed fertility outcomes in two groups of glioma survivors who had received temozolomide categorized based on whether the patient pursued fertility preservation [14]. They observed one spontaneous pregnancy in a woman who did not undergo fertility preservation and three pregnancies – one delivery, one spontaneous miscarriage, and one ongoing pregnancy – in women that underwent fertility preservation (four out of 24 women followed for one to five years). In the absence of more data, we recommend to assume high gonadotoxicity level of temozolomide, and pursuing fertility preservation in such patients following clearance by the neuro-oncologist.

A remaining concern for oncologists and oncologic surgeons is whether fertility preservation delays critical treatment. In cases when the patient receives adjuvant therapy, such as the one presented here, there is typically a sufficient interval between surgery and planned adjuvant therapy (chemotherapy and/or radiation) to allow for a short window of opportunity to freeze eggs or embryos without affecting the cancer treatment timeline at all. In addition, if neoadjuvant therapy is recommended in other cases, recent advances in reproductive technologies allows for fertility preservation to be initiated any time during the menstrual cycle (“random start”). This allows patient to start an ovulation induction cycle on the day she presents to the oncofertility specialist, and it is expected that the cycle will be no more than 2 weeks. Thus, fertility preservation should not delay or alter treatment regimens for cancer patients.

The literature is scarce regarding the possible interactions between gliomas and pregnancy. Changes of the biological behavior of some tumor subtypes may occur during pregnancy, such as an accelerated tumor growth and/or malignant transformation. Several reports have discussed interactions between pregnancy and the growth of gliomas. One study analyzed velocity of diametric expansion (VDE) of WHO grade II gliomas in 11 pregnant women and demonstrated an increase in VDE during pregnancy [4]. Multiple case series have demonstrated cases where woman with WHO grade II gliomas developed de-differentiation of the tumor during pregnancy which became apparent either clinically, radiologically or confirmed histologically by post-delivery surgeries [5–7]. A recent case report revealed a malignant transformation from diffuse astrocytoma (WHO grade II) to glioblastoma (WHO grade IV) in a post-partum patient 1 month following the patient’s delivery [8].

The mechanism by which tumor growth is enhanced during pregnancy stems from the idea that the large amount of hormones and growth factors excreted during pregnancy simultaneously increase tumor growth. Placental growth factor for example, is an angiogenic element necessary for both fetal development and the growth of gliomas [9]. Due to the relative paucity of cases reported, the majority of cases focus on gliomas diagnosed during pregnancy. There are no guidelines for the management of gliomas diagnosed either during or prior to pregnancy. If a woman with a treated glioma desires a pregnancy it is advised to perform very close neurological follow-up with repeat MRI’s in addition to obstetrical monitoring.

The use of antiepileptic drugs (AEDs) during the course of pregnancy may be teratogenic and increase the risk of congenital malformations. Levetiracetam (Keppra®) is considered a safe medication for use during pregnancy. The North American AED pregnancy registry published data collected from pregnant women taking Levetiracetam monotheraphy from 1997 to 2011. The relative risk of major malformations was not increased in comparison to women with epilepsy who did not take AEDs while pregnant [15].

To our knowledge this is the first report of successful pregnancies following fertility preservation for AA. This case illustrates the realistic possibility of oocyte or embryo cryo-preservation prior to chemotherapy and radiation with subsequent embryo transfers. A recent article published in Neuro-Oncology [16], describes a study reviewing primary brain tumor patients age 18–45, referred for fertility preservation. Seventy-three percent accepted referral to a sperm bank (87% men) or a reproductive endocrinologist (56% women). The study concludes that there is significant interest in fertility preservation among these patients, particularly if they had no children [16]. Patients should be informed at the time of tumor diagnosis about the option of preserving their fertility. Proper referral to a reproductive endocrinologist as well as a mental health professional is recommended to help make informed decisions [17].

It is incumbent upon physicians to engage in discussion of the ethical perspectives of fertility preservation in patients with brain tumors. For childless women, the option of post-treatment pregnancy opens a window of hope that may elevate their mood, helping them cope with a potentially fatal diagnosis and difficult treatment. However, the possibility that pregnancy may negatively affect prognosis remains a major concern.

## Abbreviations

AA: Anaplastic Astrocytoma
AED: Antiepileptic Drug
FET: Frozen Embryo Transfer
IDH: Isocitrate Dehydrogenase
IVF: In Vitro Fertilization
VDE: Velocity of Diametric Expansion
